# Demography Meets Climate Change: Life History Challenges for a Neotropical Viviparous Lizard

**DOI:** 10.1002/ece3.72829

**Published:** 2025-12-28

**Authors:** Maria Luiza Gonçalves Santos, Heitor Campos de Sousa, Laís Pio Caetano Machado, Guarino Rinaldi Colli

**Affiliations:** ^1^ Programa de Pós‐Graduação Em Ecologia Universidade de Brasília Brasília Brazil; ^2^ Departamento de Zoologia Universidade de Brasília Brasília Brazil

**Keywords:** ectotherm, integral projection model, mark‐recapture, population growth, survival

## Abstract

Considering the current biodiversity crisis, it is crucial to understand the impact of global environmental changes on natural populations. Analyzing demographic parameters from long‐term studies is the most effective approach to uncovering patterns that describe population dynamics. These patterns can then be linked to the environmental factors driving these dynamics, providing an accurate understanding of how environmental changes affect natural populations. This study aims to build a demographic distribution model of *Notomabuya frenata*, a Neotropical viviparous lizard, to investigate its potential responses to environmental changes. Using mark‐recapture data collected over more than 15 years, we built Integral Projection Models (IPMs) to project population trajectories across time and space based on relationships among vital rates, body size, and environmental covariates. Our work indicates that this species is positioned in the middle of the “slow‐fast” life‐history continuum of lizards, with early maturity and intermediate survivorship. We also demonstrate that it is already experiencing impacts from rising global mean temperatures, which compromise individuals' survival and ultimately reduce population growth, particularly at the northwestern periphery of its distribution. The spatially explicit approach we applied enables an understanding of the demographic consequences of climate‐induced environmental variability across different locations, recognizing that the projected impacts of climate change are unevenly distributed.

## Graphical abstract

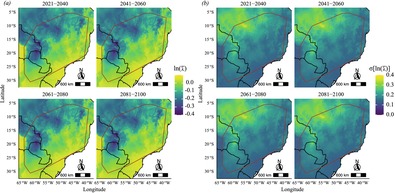

## 1 Introduction

A fundamental goal in ecology is to understand how species' life‐history traits—such as growth rates, age‐specific survival, and fecundity—interact with environmental conditions to determine spatial distribution limits (Stearns 1976). Since vital rates are environmentally dependent, the geographic range of a species can be viewed as the spatial projection of its demographic niche: the set of locations where the integration of age‐specific survival and reproduction results in population persistence (*λ* ≥ 1) (Holt 2009). Understanding these mechanistic links is particularly critical for species with complex life cycles, such as those with overlapping generations, where environmental pressures may differentially affect specific age classes (e.g., juveniles vs. reproductive adults), ultimately shaping the population's size and resilience in response to environmental changes (Jeppsson and Forslund 2012; Sæther et al. 2013). The rapid modification of climate and habitats driven by humans at local and global scales has become the primary threat to biodiversity, reflected in species' population dynamics (Cowie et al. 2022; Finn et al. 2023; Selwood et al. 2015). Notable examples of this impact include the climate‐driven extinction of the Golden Toad ( *Incilius periglenes* ), the Bramble Cay melomys ( *Melomys rubicola* ), and the widespread local extinctions of *Sceloporus* lizards across Mexico due to thermal niche collapse (Selwood et al. 2015; Sinervo et al. 2010).

Human impacts across terrestrial, freshwater, and marine ecosystems threaten more species now than ever before, with an estimated one million animal and plant species facing extinction, many within decades (IPBES 2019). This unprecedented decline affects all major vertebrate lineages, including reptiles, birds, and mammals, with particularly alarming rates observed in amphibians (> 40% threatened) and reef‐forming corals (33%) (IPBES 2019). Tropical regions, in particular, face combined risks from climate change and land‐use change (IPBES 2019). These high extinction rates are alarming because biodiversity loss reduces communities' resilience to environmental changes, destabilizing ecosystems through adverse impacts on their processes (Johnson et al. 2017; Strona and Bradshaw 2022).

Species with different life‐history strategies face distinct demographic risks under environmental changes (Jeppsson and Forslund 2012). Populations of species with fast life cycles tend to buffer adverse environmental effects through reproductive investment, while slow‐cycle species tend to invest in adult survival (Gaillard et al. 1998; Korpimäki et al. 2004). Fast‐living species are characterized by early maturity, high reproductive output, and short generation times, traits that allow populations to recover quickly from disturbances (Stearns 1976). However, this same reliance on rapid turnover also makes them more vulnerable to extreme or frequent disturbances, as such events can impact a large proportion, or even an entire cohort, before reproduction occurs. By contrast, slow‐paced species, with longer lifespans and overlapping generations, buffer the demographic effects of environmental variation across multiple reproductive events, as adult survival tends to remain stable (Gaillard et al. 1998; Koons et al. 2006). While this plasticity indicates successful adaptation to historical environmental variability (Stearns 1976), it may be insufficient to cope with rapid, directional anthropogenic climate change if thermal safety margins are exceeded (Sinervo et al. 2010). These contrasting responses are critical for predicting how life history influences population dynamics in changing environments (Sæther et al. 2013).

Thus, understanding extinction processes requires reliable predictions of population responses to environmental changes through detailed analyses of large spatio‐temporal scale datasets (Clutton‐Brock and Sheldon 2010; Gamelon et al. 2017). In this context, Integral Projection Models (IPMs) provide a powerful tool for demographic analysis. These models can integrate variations in key demographic characteristics (e.g., survival, growth, and fecundity) to describe the condition and structure of populations over time and space (Ellner and Rees 2006; Merow, Dahlgren, et al. 2014; Merow, Latimer, et al. 2014). Unlike traditional methods (matrix population models), IPMs incorporate a continuous representation of traits such as size and age, allowing for the capture of individual variation within populations (Easterling et al. 2000; Rees et al. 2014). Additionally, IPMs can integrate environmental covariates that influence these traits, providing insight into how environmental changes impact populations (Merow, Dahlgren, et al. 2014; Merow, Latimer, et al. 2014; Rees et al. 2014). This approach allows for a demographic‐level interpretation of how contemporary environmental pressures may affect species.

Ectothermic organisms, such as reptiles, are particularly vulnerable to climate change, as environmental conditions directly affect their activity times, thereby impacting their feeding, growth, and reproductive rates (Doucette et al. 2023; Gibbon et al. 2000; Paaijmans et al. 2013; Shine 2005). Global increases in atmospheric CO_2_ concentrations are driving rapid climatic changes that have already triggered local extinctions and demographic collapses in lizard populations worldwide, with viviparous species particularly vulnerable to increasing environmental temperatures (Sinervo et al. 2010). Although viviparity is hypothesized to have originated as an adaptation to cold climates (Guillette 1993; Shine 2014; Zimin et al. 2022), many clades retain this reproductive mode in warmer environments due to phylogenetic conservatism and the low evolutionary likelihood of reverting to oviparity (Blackburn 2015). Consequently, tropical viviparous species may face a climatic trap: they possess a reproductive strategy selected for heat retention, yet currently inhabit environments where rising temperatures may exceed their thermal safety margins, compromising embryonic development and imposing constraints on reproductive success and output (Jara et al. 2019; Lourdais et al. 2004). Nevertheless, more than 14.5% of reptile species still lack sufficient data to determine their threat status (IUCN 2024). Moreover, limitations related to long‐term data collection, as well as geographical and taxonomical biases, hinder the adequate understanding of ecological and demographic processes necessary to propose conservation actions and prevent local extinctions (Caetano et al. 2022; Roll et al. 2017; Winter et al. 2016).

The Scincidae family represents a globally successful radiation. Within this group, *Notomabuya frenata* (Cope 1862) belongs to the diverse and widespread subfamily Mabuyinae, a viviparous clade that successfully colonized the New World (Hedges and Conn 2012). Despite the historical evolutionary success of this lineage, ecological niche models project a severe contraction of suitable habitats for *N. frenata* populations due to climate change (Machado et al. 2023; Ribeiro‐Júnior and Amaral 2016). Small Neotropical lizards typically exhibit a fast life‐history strategy (Allen et al. 2017; Mesquita et al. 2016). *Notomabuya frenata* aligns with this pattern, achieving sexual maturity rapidly (within 2–3 months) at a small body size (49–54 mm SVL) (Vitt 1991; Vrcibradic and Rocha 1998). However, unlike oviparous species of similar size that may produce multiple clutches per season, viviparous species like *N. frenata* are constrained to a single litter per year due to the extended gestation period required for embryogenesis. This trade‐off—early maturity combined with a fixed, low reproductive frequency—suggests a specific demographic vulnerability: population stability likely relies heavily on high juvenile survival and successful recruitment during that single annual event, rendering the species particularly susceptible to climatic fluctuations that compromise specific developmental windows. Therefore, here we investigate the demographic patterns of *N. frenata* in response to environmental changes by understanding its life history and estimating the impacts of different future CO_2_ emission scenarios on its populations. To this end, we used estimates of vital rates and population structure to create a demographic distribution model for the species (Briscoe et al. 2019; Schurr et al. 2012). We predicted that increased CO_2_ emissions would lead to reduced local population growth of *N. frenata* and, eventually, to local extinctions, as the species' viviparous and ectothermic nature, combined with its fast life history, increases its vulnerability to climate change.

## 2 Methods

### 2.1 Species

*Notomabuya frenata* is a diurnal Scincidae that measures 56.7 ± 2.0 mm snout‐vent length (SVL) and forages primarily in low vegetation and shrubs, feeding on small arthropods (Vitt 1991; Vrcibradic and Rocha 1998). It ranges across the Cerrado and Chaco biomes, characterized by well‐defined dry and wet seasons and low annual temperature variation, and the Atlantic Forest, where precipitation is more evenly distributed throughout the year and temperatures are relatively stable (Nimer 1989; Vrcibradic et al. 2006). *Notomabuya frenata* is an iteroparous species with overlapping generations, capable of reproducing in multiple breeding seasons (Vitt 1991). It is viviparous, with a nine to twelve‐month gestation period, and produces litters of up to eight offspring between September and November, the beginning of the rainy season (Vrcibradic and Rocha 1998).

### 2.2 Sampling

We sampled lizards between December 2005 and January 2021, with a gap from April to July 2020 due to the COVID‐19 pandemic. The study area comprises cerrado *sensu stricto* vegetation in the *Reserva Ecológica do IBGE*, within the *Área de Proteção Ambiental das Bacias Gama e Cabeça de Veado*, Federal District, Brazil (Andrade et al. 2002; Ribeiro and Walter 2008). We conducted our study within a 50‐ha plot (Sousa et al. 2015), which encompasses a long‐term fire experiment. These different fire regimes create a heterogeneous landscape with varying levels of vegetation cover. Sampling across this area allowed us to capture population data along a representative gradient of habitat structures. We installed ten pitfall traps within each subplot, approximately 15 m apart. Traps between subplots are spaced at least 200 m apart. Each trap consists of four 35 L buckets connected by guide fences arranged in a “Y” shape. We checked traps monthly over six consecutive days. During trap checks and lizard captures, we recorded the sex, snout‐vent length (SVL) (1 mm precision ruler), and body mass (0.1 g precision scale) of individuals. We assessed the sex of individuals by palpation in females and hemipenis eversion in males. We permanently marked each captured lizard by toe‐clipping (Hero 1989) and released it at the capture site. All procedures were approved by the Animal Use Ethics Committee of the University of Brasília (process 33,786/2016).

To assess fecundity parameters, we obtained data from 30 gravid females (SVL and number of embryos) and 83 males (SVL and testes volume) of *N. frenata* housed in the Herpetological Collection from the University of Brasília. These specimens were collected during a fauna rescue operation during the filling of the Serra da Mesa reservoir (Minaçu, Goiás, Brazil); therefore, no individuals were euthanized for the present study. We obtained these data by dissecting preserved specimens, performing a direct embryo count, and measuring the testes with digital calipers. We defined the minimum size at sexual maturity based on the smallest reproductively active individuals. For females, we defined maturity by the presence of vitellogenic follicles or embryos; the smallest mature female measured 54 mm SVL. For males, we defined maturity by the presence of enlarged testes and convoluted epididymides (indicating sperm production); the smallest mature male measured 49 mm SVL. These size thresholds were used in our Integral Projection Models to define the transition from the juvenile to the adult stage, establishing the body size at which individuals begin to contribute to fertility.

### 2.3 Environmental Predictors

To model the effects of climate on demographic parameters, we sourced the environmental variables from the WorldClim database, covering the period from January 2005 to December 2020 and projections for 2021 to 2100 under two distinct socioeconomic scenarios (SSP2‐4.5 and SSP5‐8.5), each reflecting different CO_2_ emission trajectories (Calvin et al. 2023; Riahi et al. 2017). For future projections, we used data from the CMIP6 global climate model MPI‐ESM1‐2‐HR. To define the spatial extent for environmental data extraction, we compiled 251 occurrence records from the literature and herpetological collections. We used these records to construct a Minimum Convex Polygon (MCP), which served as the reference boundary for obtaining the climatic variables used in our demographic projections (Figure S1). We obtained monthly averages of maximum and minimum temperatures and precipitation at 10‐min resolution for a region encompassing the species' known range limits, with a 2‐degree buffer extending beyond the southernmost, northernmost, easternmost, and westernmost occurrence records. To prevent multicollinearity issues among predictors, we calculated each variable's variance inflation factor (VIF) and excluded those with VIF values > 5 (Shrestha 2020). This selection process resulted in retaining two variables: maximum temperature and precipitation.

### 2.4 Data Analyses

To integrate demography and distribution, we adopted a mechanistic modeling framework. We first parameterized demographic functions (survival, growth, and fecundity) based on local environmental conditions. We then utilized the spatial environmental layers (described above) to project these demographic functions across the species' range. This approach allowed us to estimate the population growth rate (*λ*) for each grid cell under present and future climate scenarios, effectively linking local demographic processes to regional distribution patterns.

We conducted all data analyses in R (version 4.3.3) (R Core Team 2024). We used the missforest package to impute 14 missing or outlier SVL values (1% of the total). To ensure accurate imputation despite caudal autotomy, the model included body mass, sex, tail length, and tail condition (intact or autotomized) as predictors (Stekhoven and Buehlmann 2012). To construct a generalized demographic model applicable to the species' distribution, we pooled data from all subplots. Because these subplots were subjected to different fire regimes, they encompass a significant gradient of vegetation structure and microclimates (Costa et al. 2020). By sampling across this environmental heterogeneity, we aimed to capture the plasticity of the species' demographic response to varying thermal environments. We assume that these physiological and demographic responses are conserved across the species' range (niche conservatism), a necessary assumption given the logistical infeasibility of obtaining long‐term demographic data at a continental scale.

Over 15 years and 2 months of monitoring, we captured and marked 1023 individuals of *N. frenata* , of which 15 were excluded from the analyses due to data inconsistencies. A total of 838 individuals were captured only once, including 282 females, 218 juveniles, 169 males, and 169 individuals with unknown sex, while 170 individuals were captured at least twice (139 individuals captured twice, 24 three times, 6 four times, and 1 five times), comprising 64 females, 15 juveniles, and 57 males, and 34 captures of individuals with unknown sex.

#### 2.4.1 Growth Model

To parameterize the growth curves for each sex, we applied the Von Bertalanffy growth curve adjusted by Fabens for mark‐recapture data, as the age at first capture was not known (Fabens 1965):

(1) $$L=L_{0}+\left(L\infty -L_{0}\right)\left(1-e^{-k*∆t}\right)$$

where *L*_*0*_ and *L* refer to the SVL of the individual at first capture and at the subsequent recapture, respectively; ∆t is the time between captures; *k* is the body growth coefficient; and *L∞* indicates the asymptotic SVL (Campbell and Phillips 1972). To capture individual variation in growth rates, we fitted a Nonlinear Mixed‐Effects Model that allowed the growth coefficient (*k*) to vary among individuals. Here, we considered 170 individuals recaptured at least once. We discarded captures of individuals with unknown sex (*n* = 58) in this analysis.

Subsequently, to estimate the age of individuals at first capture, we applied a reformulation of the original Von Bertalanffy equation, isolating age as the target variable:

(2) $$a=t_{0}+\left(\frac{1}{k}\right)\ln\left(\frac{L\infty }{\left(L\infty -L\right)}\right)$$

In this equation, *a* and *L* are the age and SVL of the individual, respectively, *t*_*0*_ is an estimated theoretical age when the individual's SVL is zero, *k* is the body growth coefficient, and *L∞* indicates the asymptotic SVL (Campbell and Phillips 1972). Using these estimates, we built a predictive growth curve that indicates the expected SVL of individuals in the population as a function of age.

To parameterize a Nonlinear Mixed‐Effects Model using the *nlme* package (Pinheiro and Bates 2000; Pinheiro, Bates, and R Core Team 2025), we defined initial estimates for the fixed (asymptotic SVL, *L* ∞) and random (body growth coefficient, *k*) effects. We assumed that the largest SVL in the dataset provides a good approximation of initial *L* ∞, and we estimated initial *k* as the mean growth rate between captures (∆SVL/∆t). We set the control parameters to 5000 iterations for the maximum number of iterations of the *nlme* optimization algorithm, 1000 iterations for the maximum number of iterations of the optimization step within the *nlme* optimization, and 0.000006 for the convergence tolerance. After running the model, we checked for homoscedasticity, model fit, and residual normality. The model explained 92% of the variation in the data, including both fixed and random effects (conditional *R* ^*2*^ = 0.92).

The Von Bertalanffy growth model assumes that SVL should never exceed *L* ∞. Therefore, we checked for any *L*_*0*_ values exceeding this limit and removed them to build the predictive growth curve, which indicates the expected SVL of individuals in the population as a function of age.

#### 2.4.2 Age‐Dependent Survival Model

To estimate age‐specific mortality rates and survival probabilities for each sex, we used a Gompertz mortality model implemented in the *Bayesian Survival Trajectory Analysis* (*BaSTA*) package (Colchero et al. 2012), which calculates survival and mortality rates from individuals' ages. To obtain the age from the individual's SVL, we applied Equation $\left(2\right)$. In this analysis, we considered 346 females, of which 28 were recaptured at least once, and 223 males, of which 38 were recaptured at least once. In the model, we included sex as a categorical variable. We set the total number of Markov Chain Monte Carlo (MCMC) steps to 50,000, the burn‐in iterations to 5001, the number of skipped MCMC steps to minimize serial autocorrelation to 50, and the number of parallel simulations to 4. The model reached appropriate convergence for all parameters.

#### 2.4.3 Environment‐Dependent Survival Model

We calculated survival probability as a function of environmental conditions for the population (characterized by overlapping generations and multiple recruitment cohorts) using a Cormack‐Jolly‐Seber (CJS) capture‐recapture model with the *marked* package (Cormack 1964; Laake et al. 2013). We combined the capture histories with monthly environmental data (mean maximum temperature and precipitation) and monthly mean SVL to create a dataset that included time‐varying covariates for each sampling occasion. For this analysis, we calculated the mean SVL for each month from December 2005 to January 2021 and selected the corresponding precipitation and maximum temperature estimates from WorldClim, extracting data specifically for the pixel covering our study area (15° 56′ 31″ S, 47° 52′ 47″ W). We therefore adjusted monthly survival in an additive model, relating it to precipitation, maximum temperature, and mean SVL variation, and then performed model selection using the Akaike Information Criterion (AIC) (Akaike 1998). We considered mean SVL variation to assess the effect of the population size structure on estimated monthly survival. This inclusion was motivated by our size‐dependent survival results (described in the results section), which indicated that mortality risk increases with body size in this population (senescence), leading to higher survival probabilities for younger classes than for older adults. We tested a set of 64 candidate models representing different additive effects of environmental covariates and mean SVL on survival and recapture probabilities. Finally, we performed a time‐series decomposition of the estimated monthly survival rates to identify which component (seasonal, trend, or residual) best explains the temporal variation in survival.

#### 2.4.4 Fecundity Model

We used Bayesian models fitted with the *brms* package to relate litter size (number of offspring) to the body length of reproductive females (Buerkner 2017). Since body size is positively correlated with age (as established by our growth models), this size‐dependent function effectively captures the increase in reproductive output associated with older, larger females. We then selected the most suitable model using leave‐one‐out cross‐validation (LOO). For each model, we ran four MCMC chains with 4000 iterations each, using 2000 warmup iterations, yielding 8000 post‐warmup samples. We applied no thinning (thinning interval = 1) and assessed convergence by inspection of trace plots. We ran several models and selected the best one based on leave‐one‐out cross‐validation.

#### 2.4.5 Population Growth Model

We constructed female‐based Integral Projection Models (IPMs) (Rees et al. 2014) to integrate the estimated vital rates (growth, survival, and reproduction) into a comprehensive demographic framework. An IPM describes the distribution of individuals of body size (SVL) *y* at time *t + 1* as follows:

$$n\left(y,t+1\right)=\int_{L}^{U}\left[P\left(x,y\right)+F\left(x,y\right)\right]n\left(x,t\right)\mathrm{dx}$$

$$=\int_{1}^{U}K\left(x,y\right)n\left(x,t\right)\mathrm{dx}$$

where *L* and *U* represent the lower and upper body size (SVL) limits, respectively. The kernel *K (x,y)* comprises two sub‐kernels: *P (x, y)*, representing survival and growth; and *F (x, y)*, representing fecundity. We implemented the IPM using the midpoint rule to discretize the continuous kernel. The body size range (*L* to *U*) was divided into 50 equally spaced intervals (mesh points), providing a resolution of approximately 1 mm.

The *P* sub‐kernel describes the probability that an individual of size *x* survives and grows to size *y* over one time step (1 month). It is calculated as the product of the survival probability (*S (x)*) and the growth transition probability (*G* (*y|x*)). We derived the growth function from the female von Bertalanffy growth model (described in Section 1). The survival function combined estimates from two sources: the size‐dependent survival model (Section 2) and the environment‐dependent CJS model (Section 3). This formulation allowed us to estimate survival as a function of both the individual's body size and the specific environmental conditions (temperature and precipitation) of each month.

The *F* sub‐kernel describes the per‐capita number of offspring produced by a female of size *x* that will enter the population at size *y*. We modeled it as the product of the probability of reproduction, litter size, and the neonate size distribution. Reproduction was restricted to the breeding season (September to January); thus, *F (x,y)* = 0 for all other months. Based on our fecundity analysis (Section 4), the effect of female SVL on litter size was not significant (95% credible interval included zero; Table S6); therefore, we assumed a constant mean litter size for all sexually mature females (SVL ≥ 54 mm). We modeled newborn size as a normal distribution based on field measurements of neonates.

To assess population viability across the species' geographic range and under future climate scenarios, we employed a spatially explicit modeling approach. Since survival rates are environmentally dependent, we constructed a specific IPM kernel for each grid cell (pixel) of the species' potential distribution for every month from 2005 to 2100. From these millions of location‐ and time‐specific kernels, we calculated the asymptotic population growth rate (*λ*) as the dominant eigenvalue of the yearly transition matrices. This metric (*λ*) served as our proxy for local population health and persistence probability. We aggregated these results to report the mean and standard deviation of *λ* for five periods: present (2005–2020) and future projections (2021–2040, 2041–2060, 2061–2080, and 2081–2100). Additionally, we conducted perturbation analyses to evaluate which demographic processes (e.g., survival, growth, reproduction) have the greatest influence on population growth. Finally, we used a time‐series decomposition of the observed monthly mean *λ* from 2005 to 2020 to determine the relative contributions of seasonality, trend, and residual to variations in population growth over time.

## 3 Results

### 3.1 Life‐History

**FIGURE 1:**
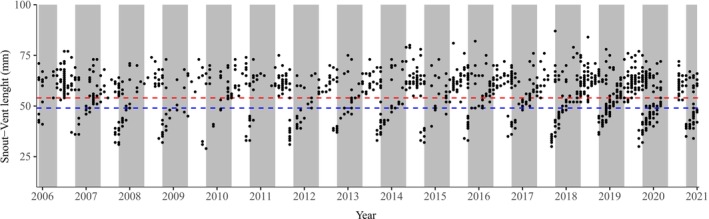
Snout‐vent length (SVL) of *Notomabuya frenata* individuals captured in Brasília, Distrito Federal, Brazil, from December 2005 to January 2021. Red and blue dashed lines represent the SVL at sexual maturity for females (54 mm) and males (49 mm), respectively. Gray bars indicate the rainy season, spanning from October to May. The y‐axis markers indicate the months of January in each year.

**FIGURE 2:**
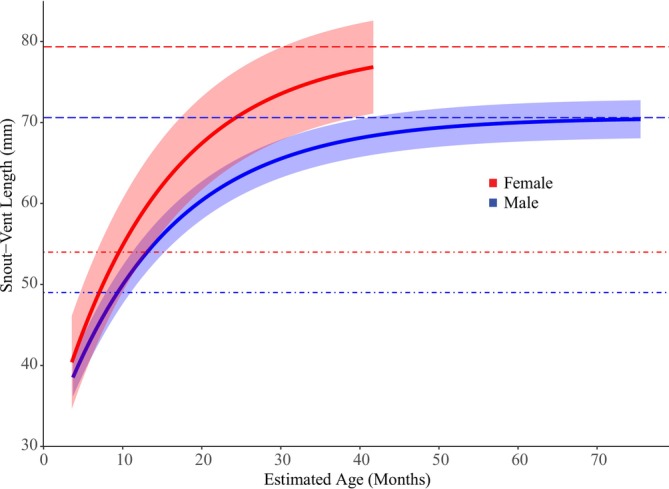
Estimated growth curve of males (blue) and females (red) of *Notomabuya frenata* captured in Brasília, Distrito Federal, Brazil, from December 2005 to January 2021. Red and blue dot‐dashed lines represent the SVL at sexual maturity for females (54 mm) and males (49 mm), respectively. Red and blue dashed lines indicate the asymptotic SVL for females (79 mm) and males (71 mm), respectively.

**FIGURE 3:**
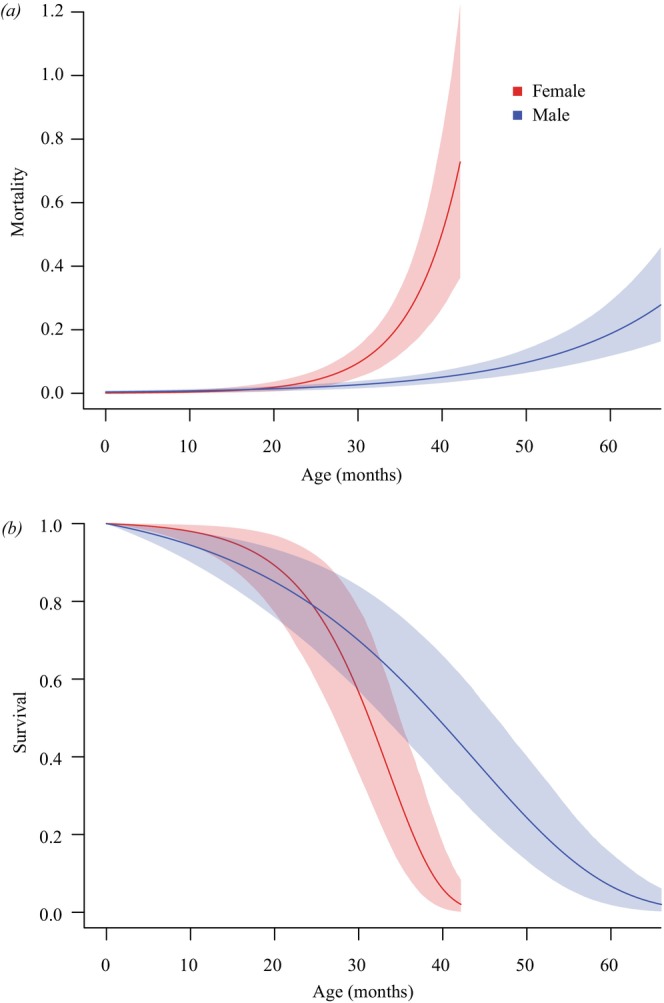
Estimated age‐specific mortality rate (a) and survival probability (b) of males (blue) and females (red) of *Notomabuya frenata* captured in Brasília, Distrito Federal, Brazil, from December 2005 to January 2021.

We detected adults year‐round, whereas juveniles were primarily observed between September and March, a period associated with the rainy season in the Cerrado (Figure 1). This recruitment pattern suggests that parturition is synchronized with the onset of rains, implying that the extended gestation period (9–12 months) encompasses the dry season. Growth models revealed sexual dimorphism in size potential: males grew slower and reached a smaller asymptotic SVL than females (Table S1 and Figure 2). The growth trajectories estimated by the models were biologically consistent with field observations: the predicted size at the beginning of the curve (age ~0) corresponds to the SVL of neonates captured during the recruitment season (~35 mm), and the inflection points align with the observed size at sexual maturity. Furthermore, the age‐dependent survival model showed that mortality patterns differed by sex (Table S2, Figure 3). Females experienced much higher mortality at older ages, while males showed a slower increase in mortality, resulting in a higher life expectancy.

**FIGURE 4:**
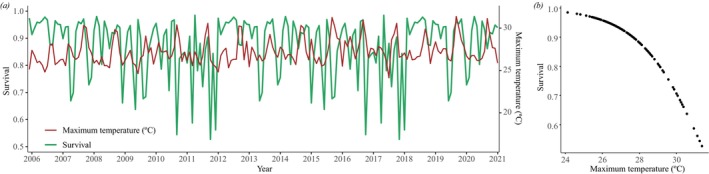
(a) Estimated monthly survival probability of *Notomabuya frenata* and maximum air temperature in Brasília, Distrito Federal, Brazil, from December 2005 to January 2021. (b) Relationship between estimated monthly survival probability and maximum air temperature.

The selected CJS capture‐recapture model (Table S3) identified an adverse effect of maximum temperature on monthly survival rates (Table S4 and Figure 4), whereas precipitation and mean SVL were not identified as important predictors of survival (Table S3). Regarding the temporal variation in monthly survival over the study period, the trend is more relevant than seasonality (Seasonality *= 0.04; Trend = 0.14*). However, we found no clear positive or negative trends over the period (Figure S2), with the residual (random effect) component being much more influential (*Residual = 0.82*).

The litter‐size model with the highest predictive performance (Table S5) showed a positive relationship between female SVL and the number of offspring (Table S6 and Figure S3). The smallest reproductive female had a snout‐vent length of 54 mm. Using Equation $\left(2\right)$, we estimated that females reach sexual maturity at 6 months. Males, in turn, reach sexual maturity at 49 mm, corresponding to 7 months of age. Based on the Integral Projection Model calculations, the mean generation time for this population is 14.5 months.

### 3.2 Population Growth

**FIGURE 5:**
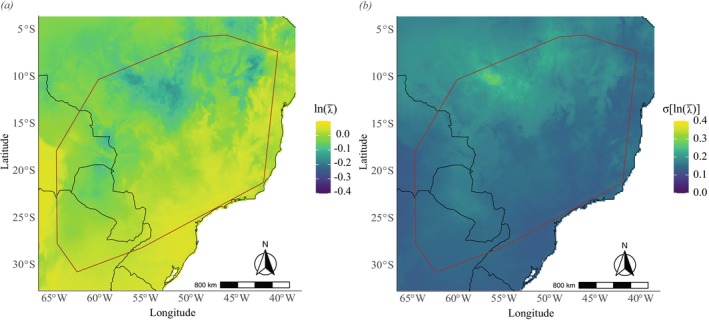
Projected mean (a) and standard deviation (b) of population growth rate (*λ*) of *Notomabuya frenata* for the period 2005–2020 on a logarithmic scale. The brown outline indicates the Minimum Convex Polygon representing the species' known distribution.

**FIGURE 6:**
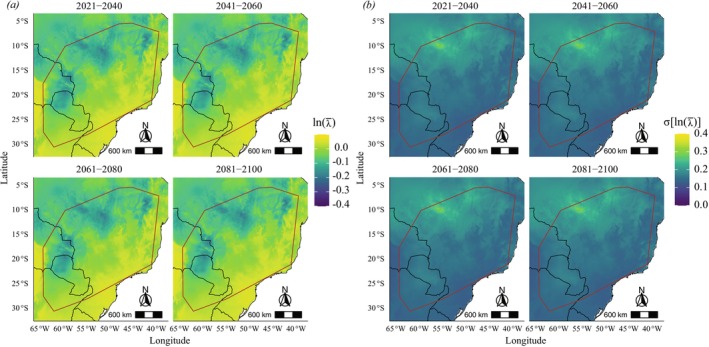
Projected mean (a) and standard deviation (b) of population growth rate (*λ*) of *Notomabuya frenata* for the period 2021–2100 under an optimistic CO_2_ emission scenario (SSP2‐4.5) on a logarithmic scale. The brown outline indicates the Minimum Convex Polygon representing the species' known distribution.

**FIGURE 7:**
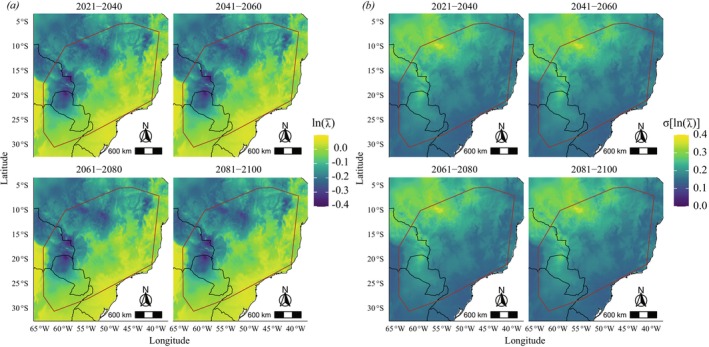
Projected mean (a) and standard deviation (b) of population growth (*λ*) of *Notomabuya frenata* for the period 2021–2100 under a pessimistic CO_2_ emission scenario (SSP5‐8.5) on a logarithmic scale. The brown outline indicates the Minimum Convex Polygon representing the species' known distribution.

**FIGURE 8:**
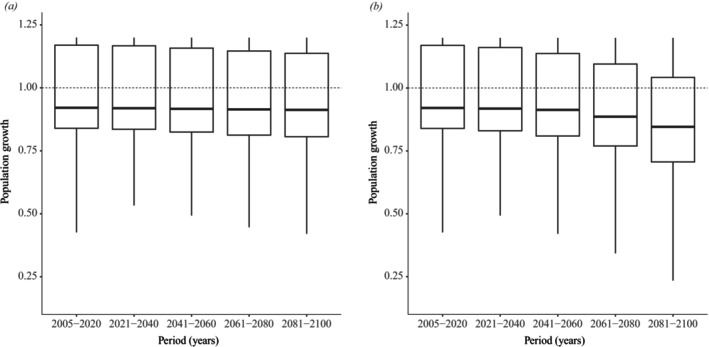
Boxplots describing the distribution of the projected mean population growth rate (*λ*) of *Notomabuya frenata* for five different periods (2005–2020, 2021–2040, 2041–2060, 2061–2080 and 2081–2100) under an optimistic CO_2_ emission scenario (SSP2‐4.5) (a) and a pessimistic CO_2_ emission scenario (SSP5‐8.5) (b).

The seasonal component accounted for 99% of the temporal variation in the population growth over the study period, with the highest *λ* values associated with the rainy season, while the trend and random (residual) components explained less than 1% of the observed variation (Figures S4 and S5). The geometric mean of *λ* for the monitored population between December 2005 and January 2021 was 1.01, indicating stability throughout the monitoring period. Sensitivity and elasticity analyses revealed that survival has a more substantial influence on population growth than fecundity (Figure S6). This result indicates that the population growth rate (*λ*) is far more sensitive to changes in survival rates than to equivalent changes in reproductive output, suggesting that the observed and projected declines in *λ* are primarily driven by the climate's negative impact on survival. By projecting the environment‐dependent demographic functions across the species' geographic range, our models indicate distinct spatial patterns in viability. Spatial projections showed that populations of *N. frenata* should experience greater growth to the south and southeast of the distribution (Figures 5a, 6a and 7a), regions where maximum temperatures are lower (Figure S7). At the same time, the standard deviation of *λ* should be slight in these regions and increase toward the northwestern periphery of the species distribution (Figures 5b, 6b and 7b). We observed changes in future projections compared to the present, with a more pronounced decline related to the high CO_2_ emission scenario (SSP5‐8.5) (Figure 8), in which *λ* values tend to decline more sharply to the northwest of the species' distribution when compared to the present or to an optimistic emission scenario (Figures 5b, 6b and 7b).

## 4 Discussion

Like other Neotropical lizards, *N. frenata* individuals present fast body growth and early maturity. Remarkably, the time to maturity (~6 months) is shorter than the gestation period (~9–12 months) (Vrcibradic and Rocha 1998). This likely reflects a strategy in which gestation serves as a “holding phase” during the resource‐poor dry season, while postnatal growth is maximized during the resource‐rich rainy season, allowing offspring to recruit into the breeding population within their first year. This pattern reflects a life‐history strategy that offsets adult mortality by maintaining a juvenile pool and enhances lifetime reproductive success by achieving early sexual maturity (Allen et al. 2017; Bonduriansky et al. 2008; James et al. 2023; Jones et al. 2014; Travers et al. 2015). Nevertheless, populations of *N. frenata* exhibit substantial overlap between generations, as their generation time is 14.5 months, while individuals can live up to 5 years. Most oviparous lizard species from the Cerrado rarely survive beyond a single reproductive cycle, that is, 1 year (Sousa et al. 2024, 2015). In sharp contrast, viviparous species such as *N. frenata* align with a medium‐ to long‐lived strategy (Tinkle 1969; Tinkle et al. 1970). This strategy effectively compensates for the reproductive constraints of viviparity: since females are physiologically limited to a single litter per breeding season, lifetime reproductive success is maximized through high adult survival and iteroparity (reproducing in multiple years) (Gunderson 1997; Recknagel and Elmer 2019). This capacity to buffer reproductive output over multiple years likely supports the species' wide distribution. Therefore, the population growth of *N. frenata* relies on a juvenile and young‐adult pool, as well as a multi‐year reproductive lifespan (potentially 4–5 breeding seasons). This combination of early maturity (fast) with iteroparity and moderate longevity (slow) places *N. frenata* in an intermediate position on the slow‐fast life history continuum of lizards (Allen et al. 2017; Mesquita et al. 2016; Stearns 1976).

Viviparity in *N. frenata* is also linked to significant intersexual divergence in demographic traits, specifically growth trajectories and mortality schedules (Ortiz et al. 2019; Vitt 1991; Vrcibradic and Rocha 1998). Females face higher body growth and mortality rates compared to males. While faster growth enables them to reach larger sizes, allowing them to carry more embryos, the added body mass and physiological changes during pregnancy might increase predation risk due to impaired mobility (Bauwens and Thoen 1981; Olsson et al. 2000; Vitt and Congdon 1978). Furthermore, viviparous females are often obligated to increase basking behavior to optimize embryonic development, a behavior that exposes them to predators precisely when their ability to escape is most compromised (Miles et al. 2000; Shine 1980). These traits can be associated with an energy trade‐off favoring reproduction (earlier sexual maturity) over longevity in females. In males, in addition to attaining sexual maturity at a smaller SVL than females, they do so later in life, reflecting lower energy allocation to growth. This pattern suggests that males prioritize energy investment in survival. Thus, while females allocate substantial energy to a single litter over an extended period, males invest in maximizing their reproductive lifespan. This strategy, supported by their higher survival rates (Figure 3), increases their cumulative lifetime opportunities to mate, potentially compensating for the prolonged gestation period of each female (Bonduriansky et al. 2008).

Compared to other lizard species across the world, *N. frenata* presents a rapid onset of sexual maturity (reaching reproductive size at ~6 months compared to a global range of 1–144 months) and intermediate longevity (5.4 years, compared to 0.9–70 years), positioning the species in the middle of the “slow‐fast” life‐history continuum of lizards (Meiri 2024; Mesquita et al. 2016). Species with early maturity and low survivorship are often exposed to demographic stochasticity and may experience significant population declines in response to disturbances (Jeppsson and Forslund 2012; Sæther et al. 2013). Conversely, they usually possess a higher potential for rapid population growth, enabling their populations to recover from declines more effectively if favorable conditions are restored (Allen et al. 2017). However, as *N. frenata* has a long generation time and small litter size, its population growth potential might be constrained by abrupt environmental changes. By doing so, in directional‐changing environments where temperatures are constantly increasing and precipitation is decreasing, *N. frenata* is left to depend on phenotypic plasticity or evolutionary adaptations (Hofmann et al. 2021; Seebacher et al. 2015).

Populations of *N. frenata* respond negatively to increased maximum temperatures, imposing substantial risks to population viability in a future with high greenhouse gas emissions. Extreme peaks in maximum temperature negatively affect the survival of *N. frenata* (Figure 4). In Cerrado, maximum temperature usually peaks at the end of the dry season, coinciding with the birth of newborns. Due to its ectothermic and viviparous traits, temperature increases could narrow the species' thermal safety margin, strongly impacting its physiological performance and activity time, and potentially impairing survival and reproduction (Huey et al. 2009; Sinervo et al. 2010). Given that population growth is largely dependent on survival, we show that global temperature increases, especially the occurrence of extreme temperature events, may jeopardize the persistence of *N. frenata* across its distribution.

In terms of population growth variation within *N. frenata* 's distribution range, the projected mean population growth rate is lower in the northwest but shows greater fluctuation (a higher standard deviation) in response to environmental variability. A high standard deviation at the periphery suggests low phenotypic plasticity in the species. However, the species persists in these areas, implying that peripheral populations may rely on metapopulation dynamics or respond differently to environmental conditions than central populations. Alternatively, our results indicate an ongoing shift toward the southeast in suitable areas, as has already been projected for other species inhabiting the Cerrado (Diniz‐Filho et al. 2012; Marini et al. 2009; Oliveira et al. 2024). If *N. frenata* 's adaptive response capacity is outpaced by the rate of climate change, the species' distribution in the northwest may contract and will continue to decrease in the coming decades (Chevin et al. 2010).

As documented in our censuses and previous studies on *N. frenata* (Machado et al. 2023; Vrcibradic and Rocha 1998), there is an overlap between the rainy season and the detection period of juveniles. In markedly seasonal environments—like those inhabited by this species—reproductive success and offspring fitness may be influenced by seasonality (Ortiz et al. 2019; Vitt and Blackburn 1991). Therefore, for young lizards that grow rapidly to achieve early sexual maturity, precipitation likely plays a more critical role in survival and growth by enhancing food availability until maturation (Ortiz et al. 2019; Vrcibradic and Rocha 1998). In the time‐series decomposition of monthly population growth rates, the seasonal component was the most significant, accounting for 99% of the variation in population growth. The highest population growth rates occurred during the rainy season, underscoring the importance of maintaining a juvenile pool for this species to remain stable. Consequently, further studies should investigate whether rapid changes in rainfall regimes and temperature might lead to phenological changes and even population declines by compromising juvenile fitness and survival.

Finally, the observed differences in energy allocation to growth and survival between sexes suggest that environmental conditions may exert distinct effects on males and females of *N. frenata* . Additionally, as a viviparous ectothermic organism, maximum temperatures critically affect the survival and population growth. Under a scenario of lower greenhouse gas emissions, we project that populations of *N. frenata* will be less impacted across its distribution. Thus, our results highlight the need to reduce greenhouse gas emissions, as the species rely on greater thermal stability. Since we considered temperature as a predictor only for survival, extinction risks may be underestimated. More detailed analyses on the reproduction constraints are therefore necessary to better understand the impacts of climate change on viviparous ectothermic populations.

## Author Contributions

**Maria Luiza Gonçalves Santos:** conceptualization (equal), data curation (equal), formal analysis (equal), investigation (equal), methodology (equal), validation (equal), visualization (equal), writing – original draft (equal). **Heitor Campos de Sousa:** conceptualization (equal), data curation (equal), formal analysis (equal), investigation (equal), methodology (equal), validation (equal), writing – review and editing (equal). **Laís Pio Caetano Machado:** conceptualization (equal), data curation (equal), formal analysis (equal), investigation (equal), methodology (equal), validation (equal), writing – review and editing (equal). **Guarino Rinaldi Colli:** conceptualization (equal), data curation (equal), formal analysis (equal), investigation (equal), methodology (equal), project administration (equal), resources (equal), supervision (equal), validation (equal), writing – review and editing (equal).

## Conflicts of Interest

The authors declare no conflicts of interest.

## Supporting information


**Data S1:** ece372829‐sup‐0001‐supinfo.docx.

## Data Availability

Data available from the Dryad Digital Repository. Reviewer sharing link: https://doi.org/10.5061/dryad.gqnk98t0k.
