# Supplementary material for: Demography Meets Climate Change: Life History Challenges for a Neotropical Viviparous Lizard
Source: Ecol Evol. 2025 Dec 28;15(12):e72829. doi: 10.1002/ece3.72829 (PMC12745924; doi:10.1002/ece3.72829)
Supplement: Supplementary file 1 — Data S1: ece372829‐sup‐0001‐supinfo.docx. [file ECE3-15-e72829-s001.docx]

**Demography meets climate change: life history challenges for a Neotropical viviparous lizard**

**Models’ parameterizations**

1. **Growth model**

**Table S1:** Output for the model describing growth trajectories of *Notomabuya frenata* in Brasília, Distrito Federal, Brazil, from December 2005 to January 2021. Values represent estimates of growth parameters for males and females. *n*: number of observations; *L*$\infty$: asymptotic snout-vent length (mm); *k*: body growth coefficient; SE: Standard Error.

| **Sex** | ***n*** | ***L***$\boldsymbol{\infty}$ **(SE)** | ***K* (SE)** |
| --- | --- | --- | --- |
| Male | 68 | 70.61 (1.22) | 0.070 (0.01) |
| Female | 60 | 79.35 (2.97) | 0.072 (0.01) |
| Juvenile | 20 | - | - |

1. **Body size-dependent survival model**

**Table S2:** Output for the age-specific mortality rate model of *Notomabuya frenata* in Brasília, Distrito Federal, Brazil, from December 2005 to January 2021. Values represent estimates of instantaneous mortality parameters for males and females. SE: Standard Error; CI: Confidence Interval.

|  | **Estimate (SE)** | **95% CI**  **(Low)** | **95% CI**  **(High)** |
| --- | --- | --- | --- |
| **Males** |  |  |  |
| Intercept | - 5.58 (0.46) | - 6.63 | - 4.82 |
| Age | 0.06 (0.01) | 0.05 | 0.08 |
| **Females** |  |  |  |
| Intercept | - 7.38 (0.87) | - 9.30 | - 5.91 |
| Age | 0.17 (0.02) | 0.12 | 0.22 |

1. **Environment-dependent survival model**

**Table S3:** Best Cormack-Jolly-Seber models (ΔAIC < 2) estimating monthly survival (*Φ*) and recapture (*p*) probabilities of *Notomabuya frenata* in Brasília, Distrito Federal, Brazil, from December 2005 to January 2021, constrained by monthly climate variables. Precip: precipitation (mm); tmax: maximum air temperature (ºC); SVL: snout-vent length (mm); AICc: Akaike information criterion corrected for small sample size.

| Model | AICc | ΔAIC |
| --- | --- | --- |
| *Φ(~ tmax) p(~ precip)* | 1827.30 | 0.00 |
| *Φ(~ tmax) p(~ tmax)* | 1827.47 | 0.17 |
| *Φ(~ tmax) p(~ tmax + precip)* | 1827.51 | 0.21 |
| *Φ(~ tmax) p(~ tmax + SVL)* | 1828.44 | 1.14 |
| *Φ(~ tmax + SVL) p(~ precip)* | 1828.80 | 1.50 |
| *Φ(~ tmax + precip) p(~ precip)* | 1829.02 | 1.72 |
| *Φ(~ tmax) p(~ 1)* | 1829.03 | 1.74 |
| *Φ(~ tmax + SVL) p(~ tmax)* | 1829.13 | 1.83 |
| *Φ(~ tmax) p(~ tmax + precip + SVL)* | 1829.29 | 1.99 |
| *Φ(~ tmax + SVL) p(~ tmax + precip)* | 1829.29 | 1.99 |
| *Φ(~ tmax) p(~ precip + SVL)* | 1829.99 | 1.99 |

**Table S4:** Output for the model describing monthly survival and recapture probabilities of *Notomabuya frenata* in Brasília, Distrito Federal, Brazil, from December 2005 to January 2021, constrained by monthly climate variables. Values represent estimates of survival and recapture parameters and covariates of the Cormack-Jolly-Seber model *Φ(~ tmax) p(~ precip)*. *Φ*: probability of survival, *p*: probability of capture. Precip: precipitation (mm); tmax: maximum air temperature (ºC); SE: Standard Error; CI: Confidence Interval.

|  | Estimate (SE) | 95% CI  (Low) | 95% CI  (High) |
| --- | --- | --- | --- |
| *Φ* |  |  |  |
| Intercept | 17.73758 (3.79660) | 10.29622 | 25. 17893 |
| tmax | - 0.56193 (0.13309) | - 0.82279 | - 3.01067 |
| *p* |  |  |  |
| Intercept | - 3.65153 (0.14159) | - 3.92905 | 3.37400 |
| precip | - 0.00157 (0.00083) | - 0.00319 | 0.00005 |

1. **Reproduction model**

**Table S5:** Models describing litter size of *Notomabuya frenata* in Minaçu, Goiás, Brazil, constrained by female snout-vent length (SVL). *n*: litter size; elpd: expected log predictive density; SE: Standard Error; *s*: defines a non-linear model with smoothed spline.

| Model | elpd (SE) | $\boldsymbol{\Delta}$elpd |
| --- | --- | --- |
| *n(~ SVL)* | -52.1 (1.1) | 0.0 |
| *n(~1)* | -52.6 (1.0) | -0.5 |
| *n(~s(SVL))* | -52.6 (1.1) | -0.5 |
| *n(~*$\boldsymbol{SVL}^{\boldsymbol{2}}$*)* | -52.9 (1.2) | -0.8 |

**Table S6:** Output for the model describing litter size of *Notomabuya frenata* in Minaçu, Goiás, Brazil, constrained by female snout-vent length. Values represent estimates of reproduction parameters of the *brms* model *~ SVL*. SVL: snout-vent length; SE: Standard Error; CI: Confidence Interval.

|  | Estimate (SE) | 95% CI  (Low) | 95% CI  (High) |
| --- | --- | --- | --- |
| Intercept | - 0.26 (1.08) | - 2.37 | 1.82 |
| SVL | 0.02 (0.02) | - 0.01 | 0.06 |

**Figures**

**Figure S1:** 251 occurrence records of *Notomabuya frenata* (black dots) and the Minimum Convex Polygon (outline) delineating the species’ known distributional range.

**Figure S2:** Decomposition of the time series of estimated monthly survival of *Notomabuya frenata* in Brasília, Distrito Federal, Brazil, from December 2005 to January 2021. Seasonal effect: 0.04; trend effect: 0.14; remainder effect: 0.82.

**Figure S3:** Relationship between brood size and female snout-vent length (SVL) in adult *Notomabuya frenata* at Minaçu, Goiás, Brazil.

**Figure S4:** Estimated monthly population growth (*λ*) of *Notomabuya frenata* in Brasília, Distrito Federal, Brazil, from December 2005 to January 2021. Gray bars indicate the rainy season, spanning from October to May. The y-axis markers indicate January of each year.

**Figure S5:** Decomposition of the time series of estimated monthly population growth (*λ*) of *Notomabuya frenata* in Brasília, Distrito Federal, Brazil, from December 2005 to January 2021. Seasonal effect: 0.9986; trend effect: 0.0003; remainder effect: 0.0011.

**Figure S6:** Discretized IPM mean kernel (a), sensitivity (c) and elasticity (b) from 2005 to 2020, derived from combining growth, survival and fecundity models, depicted as a heat map. The x-axis represents snout-vent length at time *t* and the y-axis represents snout-vent length at time *t+1*, both in millimeters.

**Figure S7:** Historical mean maximum temperature for the period 2005-2020.

**References**

Buerkner, P.-C. (2017). brms: An R package for Bayesian multilevel models using Stan. *Journal of Statistical Software*, *80*(1), 1–28. https://doi.org/10.18637/jss.v080.i01

Colchero, F., Jones, O. R., & Rebke, M. (2012). BaSTA: An R package for Bayesian estimation of age-specific survival from incomplete mark-recapture/recovery data with covariates. *Methods in Ecology and Evolution*, *3*(3), 466–470. https://doi.org/10.1111/j.2041-210X.2012.00186.x

Laake, J. L., Johnson, D. S., & Conn, P. B. (2013). marked: An R package for maximum likelihood and Markov Chain Monte Carlo analysis of capture-recapture data. In *Methods in Ecology and Evolution* (Vol. 4, Issue 9, pp. 885–890). WILEY. https://doi.org/10.1111/2041-210X.12065

Pinheiro, J., Bates, D., & R Core Team. (2025). *Nlme: Linear and Nonlinear Mixed Effects Models. R package version 3.1-168*. https://doi.org/10.32614/CRAN.package.nlme
